# Laparoscopic Cytoreductive Surgery and HIPEC in Patients with Limited Pseudomyxoma Peritonei of Appendiceal Origin

**DOI:** 10.1155/2012/981245

**Published:** 2012-04-11

**Authors:** Jesus Esquivel, Andrew Averbach

**Affiliations:** Department of Surgical Oncology, St. Agnes Hospital, Baltimore, MD 21229, USA

## Abstract

*Introduction*. Increasing numbers of patients with pseudomyxoma peritonei (PMP) of appendiceal origin are being evaluated with a low tumor burden. We explored a minimally invasive approach for this group of patients. *Materials and Methods*. We designed a protocol in which patients with a PMP diagnosis would have a diagnostic laparoscopy. If limited carcinomatosis (PCI ≤ 10) is identified, the procedure will continue laparoscopically. If extensive carcinomatosis (PCI > 10) is found, then the procedure will be converted to an open approach. *Results*. From December 2008 to December 2011, 19 patients had a complete cytoreduction and HIPEC: 18 of them (95%) were done laparoscopically and 1 of them (5%) was converted to an open procedure. Mean PCI was 4.2. Grade 3 morbidity was 0, and one patient (5%) experienced a grade 4 complication, needing a reoperation for an internal hernia. There were no mortalities. Mean length of hospital stay was 5.3 days. At a mean follow-up of 17 months (1–37) all 19 patients are alive and free of disease. *Conclusion*. This study demonstrates that cytoreductive surgery and HIPEC via the laparoscopic route is feasible and safe and should be offered to patients with limited pseudomyxoma peritonei of appendiceal origin.

## 1. Introduction

Cytoreductive surgery (CRS) and hyperthermic intraperitoneal chemotherapy (HIPEC) have become standard of practice in patients with pseudomyxoma peritonei [1]. The open procedure has been associated with grade III and IV morbidity and prolonged hospitalization. In addition, many patients with PMP are being referred to a peritoneal surface malignancy center as soon as they are diagnosed and not after 3 or 4 abdominal procedures as we used to see in the late 90s. Furthermore, the number of patients that are diagnosed after a laparoscopic appendectomy is on the rise as well. What to do in this particular group of patients is still a matter of debate, with half of the cytoreductive surgeons recommending a watch-and-wait approach and the other half recommending cytoreductive surgery and HIPEC. One of the problems with the watch-and-wait approach is that it generates anxiety in some patients and that the followup requires numerous CT scans. This of course exposes the patient to increasing doses of radiation. MRI is becoming a very useful tool to evaluate the abdomen and pelvis for additional mucinous implants and hopefully will help to reduce the amount of radiation exposure. An obvious disadvantage of treating every patient with PMP with a very large incision in order to rule out the presence of any residual disease is the fact that many of these patients are going to have very limited peritoneal disease. Therefore, this approach represents an opportunity to improve patient care. In this modern era of individualized medicine, reports on laparoscopic surgery for cancer patients have been published in just about every organ in the abdomen [2–4]. Prospective randomized trials have shown that there is no difference in port site and wound recurrence, no difference in distant recurrence, and no difference in survival in patients undergoing laparoscopic surgery for primary colon cancer, in fact, some of these studies show a better outcome in those having laparoscopic surgery [5].

For these reasons, and understanding that laparoscopic surgery is *not* a different surgery but rather just a different approach, our group decided to evaluate the role of laparoscopic cytoreductive surgery and HIPEC in patients with limited peritoneal dissemination. The results with the first 14 patients that included a variety of peritoneal surface malignancies look very promising and have been previously published [6]. The purpose of this paper is to report our continued experience from this protocol in patients with pseudomyxoma peritonei of appendiceal origin, also referred to as low-grade mucinous carcinoma peritonei (L-MCP) or disseminated peritoneal adenomucinosis (DPAM).

## 2. Materials and Methods

**Figure 1:**
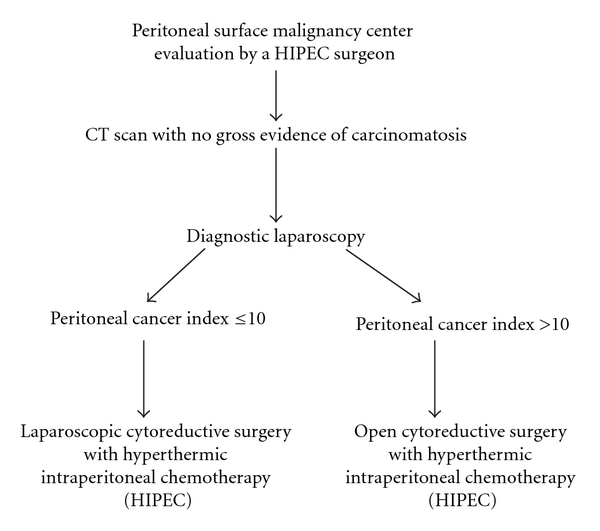
Clinical pathway for the laparoscopic management of peritoneal surface malignancies.

Patients with a histological diagnosis of low-grade, mucinous carcinoma peritonei of appendiceal origin and no gross evidence of carcinomatosis on the CT scan were subjected to a diagnostic laparoscopy to determine the peritoneal cancer index. During laparoscopy, if low-volume carcinomatosis is identified, defined as a Peritoneal Cancer Index (PCI) ≤ 10, the oncological procedure will be continued laparoscopically. If at the time of the diagnostic laparoscopy high-volume carcinomatosis is identified, defined as a PCI > 10, the procedure will be converted to the standard open approach. The protocol (RPN 2008-020) was approved by our Institutional Review Board (Figure 1).

**Figure 2:**
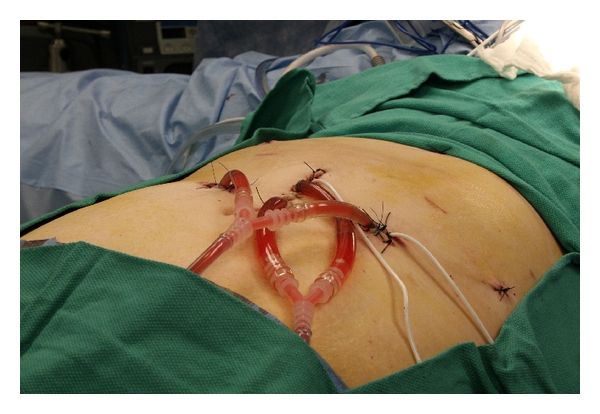
Laparoscopic hyperthermic intraperitoneal chemotherapy (HIPEC).

The operative team consisted of a very experienced laparoscopic surgeon (A. Averbach) who normally performs more than 150 complex laparoscopic bariatric operations a year and a cytoreductive surgeon (J. Esquivel). The procedures were performed in an integrated minimally invasive operating room with STORZ high-definition laparoscopic equipment. The peritoneal cavity was accessed in the right-upper quadrant with a direct view 12 mm Visiport with a 0 degree laparoscope. Once the pneumoperitoneum was established, two additional 12 mm trocars were placed in the periumbilical and left-upper quadrants under direct visualization. Two 5 mm trocars were placed below the right and left costal margins. Occasionally, a sixth trocar (5 mm) was placed in the midline above the symphysis pubis to facilitate upper abdomen dissections. Lysis of adhesions was performed to free all intra-abdominal structures, and a detailed exploration of both parietal and visceral peritoneum was carried out in order to determine the laparoscopic Peritoneal Cancer Index (PCI). This was facilitated by instrument retraction and positioning the operating room table for gravity-assisted retraction in order to maximize exposure of dependent areas. Once the diagnostic laparoscopy was completed, a decision to continue with the cytoreduction via the laparoscopic route was made based on two factors: the PCI had to be 10 or less and the amount of disease present had to be able to be removed laparoscopically according to the senior surgeon (A. Averbach). We decided on a PCI of 10 or less as this amount of carcinomatosis is what we normally consider as low-volume carcinomatosis in all peritoneal surface malignancies. All patients underwent a greater omentectomy even if there was no evidence of macroscopic disease. The greater omentum was mobilized off the transverse colon, and its hepatic and splenic flexures were taken down for complete excision using the Harmonic scalpel (Ethicon Inc., Guaynabo, PR). The gastrosplenic ligament was severed close to the splenic hilum. Additional visceral resections and peritoneal stripping were performed as needed in order to achieve a complete cytoreduction. Bowel resections were performed with an Endo GIA 3.5/60 mm cartridge (US Surgical, Norwalk, CT) and the staple lines inverted with a running 2.0 Surgidac Endostitch (US Surgical, Norwalk, CT) when needed. The bowel mesentery was transected with the Harmonic scalpel (Ethicon Inc., Guaynabo, PR). Bilateral salpingo-oophorectomies were done with suture ligation of the origins of the Fallopian tubes and subsequent dissection with the Harmonic scalpel (Ethicon Inc., Guaynabo, PR). At the end of the laparoscopic stage of the procedure, a 6 cm periumbilical midline laparotomy was performed and the specimens were extracted. Two inflow and 2 outflow perfusion catheters were placed, and the skin at the laparotomy and port sites was closed with a running nylon stitch to avoid chemotherapy solution leakage and to expose all incisions to its action to reduce the risk of tumor cell implantation. Hyperthermic intraperitoneal chemotherapy with 40 mg of Mitomycin C for 90 minutes at 43 degrees Celsius was administered using either the Belmont (Belmont Instruments, Billerica, MA) or ThermaSolutions (Thermasolutions Inc., Pittsburgh, PA) perfusion systems (Figure 2). At the completion of the heated perfusion, gastrointestinal anastomosis was performed as indicated, the midline laparotomy incision was closed with a running looped 1.0 Maxon stitch (US Surgical, Norwalk, CT), the trocars were reinserted, and the peritoneal cavity was explored to assure lack of visceral injuries and/or bleeding sources. The 12 mm port site incisions were closed with 0 POLYSORB (US Surgical, Norwalk, CT) with a Carter-Thomason suture closing system (Cooper Surgical, Trumbull, CT). In the patient in whom the procedure was converted to an open intervention, the same principles were followed: resections were made, the chemotherapeutic perfusion was carried via the closed abdomen method, and after the perfusion the abdomen was closed with a running looped 1.0 Maxon stitch (US Surgical, Norwalk, CT). Postoperative complications were reported according to the National Cancer Institute Common Toxicity Criteria. Follow-up included a CEA and CA 19-9 every 3 months, a baseline postoperative CT scan at 4 months and then a repeat CT scan or MRI every 6 months.

## 3. Results

From December 2008 to December 2011, 30 patients with limited peritoneal surface malignancies were taken to the operating room for a laparoscopic cytoreductive surgery and HIPEC. Of these 30 patients, 19 patients had the diagnosis of Pseudomyxoma Peritonei of appendiceal origin and constitute the basis of this study. The most common form of presentation of their appendiceal mucinous neoplasm was an ovarian mass with nearly half of the female patients, 6 out of 14 presenting this way (Table 1). Thirteen patients were enrolled into the protocol with 8 of them previously reported in another manuscript [6]. Once the protocol was completed, we included 6 more patients done off-protocol. There were 15 females and 4 male patients. Mean age was 52 (38–74). All patients had previous surgeries. Sixty-six percent had a previous laparoscopic procedure and 33% a previous open procedure. Median time from initial surgery to cytoreduction and HIPEC was 3 months (1–22). All 19 patients had a complete cytoreduction and HIPEC; 18 (95%) were done laparoscopically and 1 (5%) was converted to an open procedure because the evaluation by the senior surgeon (A. Averbach) indicated that we would not be able to remove the disease that was present in the previous anastomosis via the laparoscopic route. This case, which was the third patient on the study, is the only patient with PMP that was converted to an open procedure, and it happened 33 months ago; since then, the following 16 cases have been completed laparoscopically. The mean PCI was 4.3 (1–10), and mean operative time was 4.2 hours (3.5–6) (Table 2). Forty-four percent of the patients required a limited peritonectomy, and 27% required a bowel resection (Table 3). Mean blood loss was 50 mL, and no patients received a blood transfusion. All patients were extubated at the end of the procedure and transferred to the postanesthesia care unit and then to the regular surgical ward. No nasogastric tubes or intraabdominal drains were placed. Grade 3 morbidity was zero, and one patient (5%) in the laparoscopy group experienced a grade 4 complication, needing a reoperation for an internal hernia; this reoperation was also completed laparoscopically, and the patient went home 14 days after the first surgery. There were no operative deaths. Mean length of hospital stay was 5.3 days (3–14). A summary of pathological findings is included on Table 4. At a mean followup of 17 months (1–37), all patients are alive and well, with no evidence of disease recurrence.

## 4. Discussion

The first case report of cytoreductive surgery and HIPEC in a patient with Pseudomyxoma Peritonei (PMP) of appendiceal origin dates back to 1979 [7]. Since then, cytoreductive surgery and HIPEC have become the standard treatment for this group of patients even though there has never been a prospective randomized trial. Analysis of the published data demonstrates that a complete surgical eradication of this low-grade type of tumor is associated with the best outcome, and while the added benefit of the hyperthermic intraperitoneal chemotherapy to a complete cytoreduction continues to be a matter of debate and has never been clearly established, a trial that would compare cytoreductive surgery with or without HIPEC in patients with PMP is just not a feasible trial.

When it comes to the mode of presentation of PMP of appendiceal origin, nothing has changed too much in the last 3 decades. An ovarian mass continues to be a very common presentation in women, as well as being diagnosed after an appendectomy for appendicitis. What has changed is the amount of tumor burden. An increasing abdominal girth used to be a very common presentation [8], and now it is becoming a rather infrequent one. The treatment remains the same: cytoreductive surgery to remove all visible tumor and HIPEC to eradicate microscopic residual disease.

Recommending surgery in patients with Pseudomyxoma peritonei after their initial diagnosis has been established but now having a negative CT scan on follow-up remains a topic of discussion. There appears to be an unwritten agreement on what to do with patients that have no gross evidence of carcinomatosis but that have epithelial cells outside of the appendix. Most cytoreductive surgeons will recommend cytoreductive surgery and HIPEC. In this series, 72% of the patients had epithelial cells outside of the appendix and extracellular mucin was found in 100% of the patients.

We used to recommend a watch-and-wait followup for patients that had a perforated appendix, had a negative CT scan, and only mucin in the periappendiceal tissue. We had believe that most of these patients will not go on to develop pseudomyxoma peritonei syndrome. We were concerned with the amount of radiation exposure as a result of multiple CT scans, and for this reason now we use MRI to follow these patients. However, some of these patients do not feel comfortable with just a watch-and-wait approach, and we do not believe that a patient should have an exploratory laparotomy to document that they do not have any disease. This was the initial rationale for developing a minimally invasive approach. It is interesting that in this study, of the 16 omentums that looked normal even during the laparoscopic examination, 81% had at least mucin found during the pathological examination. Of course we do not know what would be the natural history of that finding, but it is a finding, that raises a valid concern.

Our current approach and recommendations are as follows: if the patient has a ruptured mucinous appendiceal neoplasm, has a CT scan or MRI with no gross evidence of peritoneal dissemination, and has epithelial cells in the periappendiceal tissue, we recommend a laparoscopic cytoreductive surgery that includes a greater omentectomy, a portion of the lesser omentum, a bilateral salpingo-oophorectomy in postmenopausal women or premenopausal women that do not wish to have children, and HIPEC. If the patient has a ruptured mucinous neoplasm with only mucin in the peri-appendiceal tissue, we recommend a peritoneal metastases protocol MRI and if that does not show any evidence of mucinous deposits, then we recommend follow-up with a routine MRI every 6 months for the first 3 years and then once a year.

It is important to emphasize that this approach needs longer followup, and as any new therapeutic approach, it should be done under the auspices of a clinical research protocol. In order to decrease the learning curve, the surgical team should include not only a cytoreductive surgeon but a surgeon that does minimally invasive surgeries on a routine basis. As mentioned before, our only conversion was 33 months ago this represented our 3rd case, and the patient that needed the reoperation was our second patient. We have learned since then that the minimally invasive nature of early Pseudomyxoma Peritonei is amenable to a minimally invasive management and treatment.

## 5. Conclusion

This initial investigative stage demonstrates that laparoscopic cytoreductive surgery and HIPEC in patients with limited peritoneal dissemination from Pseudomyxoma Peritonei of appendiceal origin are feasible and safe and therefore should be added to the armamentarium of treatment options for this group of patients.

## Figures and Tables

**Table 1:** Mode of presentation in 18 patients with PMP.

Event	Number	Percentage
Ovarian mass	6	33
Abdominal Pain/CT scan	6	33
Appendicitis	3	16
Other	3	16

**Table 2:** Characteristics of patients with limited pseudomyxoma peritonei (L-MCP) treated with cytoreductive surgery and HIPEC.

Variable	Laparoscopic CRS and HIPEC	Laparoscopic to open CRS and HIPEC
Number of patients (*n*)	18	1
Mean age	50	74
Sex		
Male	4	0
Female	14	1
Previous surgery		
No	0	0
Yes	18	1
Previous chemotherapy		
No	18	1
Mean body mass index	26.2	29.1
Mean peritoneal cancer index	4.2	10
Complete cytoreduction		
Yes	18	1
Bowel resection		
No	13	1
Yes	5	0
Mean estimated blood loss	50 mL	100 mL
Mean blood transfused	0	0
Mean duration of surgery	4.2 hours	5 hours
Grade 3 complications		
No	18	1
Grade 4 complications		
No	17	1
Yes	1	0
Mean length of hospital stay	5.3 days	7 days
Mean follow-up	17 months	33 months

**Table 3:** Procedures performed in 18 patients undergoing laparoscopic cytoreduction.

Surgical resection	Number	Percentage
Greater omentectomy	17	94
Limited peritonectomy	8	44
Bowel resection	5	27
Salpingo-oophorectomy	2	11

**Table 4:** Summary of pathological findings.

Event	Number	Percentage
Extracellular mucinous deposits		
outside of the appendix	18	100
Epithelial cells present		
outside of the appendix	13	72
Omentum with grossly		
normal appearance	16	89
Final pathology positive	13	81
Pathology of the omentum		
showing extracellular mucin	10	62
Pathology of the omentum		
showing epithelial cells	3	18
